# Old flies have a robust central oscillator but weaker behavioral rhythms that can be improved by genetic and environmental manipulations

**DOI:** 10.1111/j.1474-9726.2012.00800.x

**Published:** 2012-06

**Authors:** Wenyu Luo, Wen-Feng Chen, Zhifeng Yue, Dechun Chen, Mallory Sowcik, Amita Sehgal, Xiangzhong Zheng

**Affiliations:** 1 Cell and Molecular Biology Program, University of Pennsylvania Perelman School of MedicinePhiladelphia, PA, USA; 2 Howard Hughes Medical Institute, University of Pennsylvania Perelman School of MedicinePhiladelphia, PA, USA; 3 Department of Neuroscience, University of Pennsylvania Perelman School of MedicinePhiladelphia, PA, USA

**Keywords:** aging, behavioral rhythms, circadian clock, period, protein kinase A, sleep

## Abstract

Sleep–wake cycles break down with age, but the causes of this degeneration are not clear. Using a *Drosophila* model, we addressed the contribution of circadian mechanisms to this age-induced deterioration. We found that in old flies, free-running circadian rhythms (behavioral rhythms assayed in constant darkness) have a longer period and an unstable phase before they eventually degenerate. Surprisingly, rhythms are weaker in light–dark cycles and the circadian-regulated morning peak of activity is diminished under these conditions. On a molecular level, aging results in reduced amplitude of circadian clock gene expression in peripheral tissues. However, oscillations of the clock protein PERIOD (PER) are robust and synchronized among different clock neurons, even in very old, arrhythmic flies. To improve rhythms in old flies, we manipulated environmental conditions, which can have direct effects on behavior, and also tested a role for molecules that act downstream of the clock. Coupling temperature cycles with a light–dark schedule or reducing expression of protein kinase A (PKA) improved behavioral rhythms and consolidated sleep. Our data demonstrate that a robust molecular timekeeping mechanism persists in the central pacemaker of aged flies, and reducing PKA can strengthen behavioral rhythms.

## Introduction

A prominent problem among the elderly is that of disrupted sleep–wake cycles (Duffy & Czeisler, 2002). The mechanisms underlying this disruption are not understood, although decrements in circadian clock function are a distinct possibility. While there is no age-related loss of neurons in the central pacemaker, the suprachiasmatic nucleus (SCN) in hypothalamus (Madeira *et al.*, 1995), the amplitude of the electrical activity rhythm is reduced in older animals (Satinoff *et al.*, 1993; Watanabe *et al.*, 1995; Aujard *et al.*, 2001). This weakening of rhythm strength is thought to arise from decreased amplitude of individual SCN neurons and increased variability among neurons (Aujard *et al.*, 2001). Transplantation of a fetal SCN into aged animals restored circadian rhythms in levels of hypothalamic corticotrophin-releasing hormone (CRH) mRNA, supporting the idea that the central pacemaker is a target of the aging process (Cai *et al.*, 1997). However, the effect of aging on the molecular clock within SCN neurons is unclear. Some studies reported reduced expression of specific clock genes in the aged SCN, although the genes affected may vary by species (Weinert *et al.*, 2001; Kolker *et al.*, 2003), but other studies found robust cycling of circadian clock gene transcription (Asai *et al.*, 2001). In addition, expression of a *per1*-luciferase reporter is robust in the aged rat SCN although the free-running period is significantly shorter (Yamazaki *et al.*, 2002), while *per2*-luciferase reporter cycling is dampened in the SCN of middle-aged mice (Nakamura *et al.*, 2011). At the protein level, age affects the amplitude and/or phase of expression of the CLOCK and BMAL1 clock proteins in several extra-SCN regions, but not in the mouse SCN (Wyse & Coogan, 2010). A recent study found minor deficits of PER2 expression in the SCN of middle-aged mice (Nakamura *et al.*, 2011).

Given that the SCN consists of tens of thousands of heterogeneous neurons, it is possible that a small population of SCN neurons is sufficient to affect locomotor rhythms (Pendergast *et al.*, 2009), and age-related changes in these neurons are not always sampled in preparations using SCN explants or sections. In contrast, the circadian pacemaker network in the *Drosophila* brain consists of distinct groups of clock neurons totaling approximately 150 (see review in Nitabach & Taghert, 2008). The distinct spatial organization of the *Drosophila* circadian oscillator provides an ideal system to address potential age-related defects in the circadian timekeeping network.

To determine whether circadian deregulation contributes to the aging phenotype, we systematically analyzed flies of different age groups for deficits in the circadian system: rest–activity behavior in free-running conditions and cyclic expression of clock gene products in peripheral and central clocks. We report here that middle-aged flies display longer circadian periods, and older flies, particularly females, show an unstable rest–activity pattern that eventually degenerates into arrhythmia. While circadian expression of clock genes in peripheral tissues declines, molecular oscillations of the central clock are robust and also synchronized among the clock network, even in arrhythmic flies at the terminal life stage. Interestingly, manipulations of environmental conditions, such as coupling the light–dark cycle with a temperature cycle, or reduction in protein kinase A (PKA), improve the strength of behavioral rhythms.

## Results

### Circadian behavioral rhythms deteriorate as a function of age

The isogenic *w*^1118^ (iso31) strain used in this study lives up to approximately 90 days on standard culture medium when reared under 12:12 h light–dark (LD) cycles at 25 °C (Koh *et al.*, 2008). To specifically assess the impact of aging on free-running circadian rhythms (those driven by a circadian clock in the absence of environmental cues), we monitored the locomotor activity of individual flies at different ages in constant darkness (DD). Five age groups were examined: young (5–8 days), middle-aged (40–43 days), old (two subgroups of 53–57 days and 69–71 days) and those in the terminal life stage (79–80 days).

**Fig. 1:**
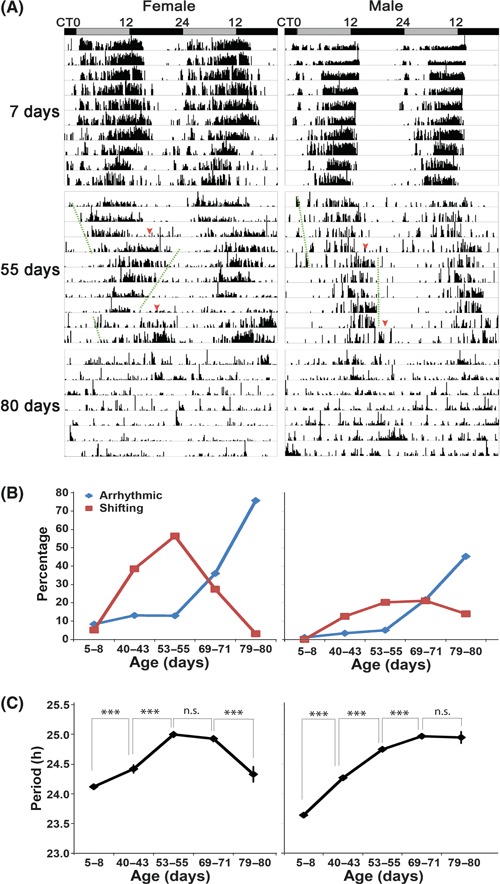
Characteristics of circadian behavior in young and old flies. (A) Locomotor behavior was assayed in constant darkness for young (7 days) and old flies (55 days and 80 days) and representative activity records are shown. In addition to having a longer circadian period, 55-day-old flies display an unstable activity pattern that consists of periodic changes in the phase of activity (middle panel, dashed lines indicate segments with overt periods and arrowheads indicate phase-shifting). Shifting flies are those rhythmic flies showing an abrupt, more than 1 h phase shift of their activity offset after a few days in DD. Most of these flies also show a more than 0.5 h change of circadian period after the shift. The shifting and instability of period phenotypes are more pronounced in females than in males. Representative activity records of arrhythmic terminal-aged (80 days) flies are shown in the lower panel. The gray bar denotes subjective day, and the black bar denotes subjective night. (B, C) Age affects circadian rhythmicity and period. (B) Higher proportions of old flies exhibit unstable (shifting) and arrhythmic behavior. (C) Circadian period lengthens with age. Period was calculated by chi-square periodogram analysis for those rhythmic flies (including those that have shifting and non-shifting behavior) and shown as average period ± standard error of mean (SEM) for each age group. ***P < 0.001, and n.s. denotes no significant difference by Student’s *t*-test. Also see Fig. S1 and Table 1 for details.

While young flies (5–8 days) have robust rest–activity rhythms, most aged flies show long period and a significant portion of them display an unstable behavioral pattern, which includes instability of phase and increased arrhythmicity (Figs 1 and S1, Table 1). More female flies display these changes than do males in all age groups. Interestingly, some aged flies show shifts in the phase of activity in a consistent fashion within each gender: every 4–5 days for females and 5–6 days for males (Figs 1A and S1). This shift is also accompanied by a change of circadian period, especially in some aged females: while these flies have a longer circadian period for the first few days in DD, a shorter circadian period follows the shift. However, the apparent change of period may result from a change in activity distribution, or a change in the length of the active phase (see Discussion). The overall circadian period (tau), measured over an extended period of time in DD, is longer in aged flies, with the exception that rhythmic female flies at the terminal life stage have a period length comparable to that of young flies (Fig. 1C, Table 1). Because most flies are arrhythmic at this stage, those that are rhythmic may be relatively resistant to effects of age on the circadian system, therefore the normal period.

**Table 1:** Characterization of circadian behavior of aging flies

	Age (day)	5–8	40–43	53–55	69–71	79–80
Female						
DD	*n*	96	122	170	186	95
	Period (h)	24.12 ± 0.04	24.42 ± 0.07	25.00 ± 0.05	24.93 ± 0.05	24.33 ± 0.13
	FFT	0.043 ± 0.003	0.039 ± 0.002	0.041 ± 0.002	0.040 ± 0.002	0.034 ± 0.006
	AR (%)	8.3	13.1	12.9	36.0	75.8
	Shifting (%)	5.2	38.5	56.5	27.4	3.2
LD	*n*			85	95	138
	Period (h)			23.91 ± 0.03	23.97 ± 0.08	23.87 ± 0.07
	AR (%)			29.4	48.4	59.4
Male						
DD	*n*	94	119	198	175	86
	Period (h)	23.64 ± 0.03	24.27 ± 0.04	24.75 ± 0.03	24.97 ± 0.04	24.95 ± 0.10
	FFT	0.120 ± 0.004	0.057 ± 0.003	0.054 ± 0.002	0.039 ± 0.002	0.038 ± 0.004
	AR (%)	1.1	3.4	5.1	21.7	45.4
	Shifting (%)	0	12.6	20.2	21.1	14.0
LD	*n*			97	116	90
	Period (h)			23.94 ± 0.02	23.91 ± 0.03	24.00 ± 0.05
	AR (%)			29.8	50.0	60.0

Total number (*n*) includes individual flies that survived more than 5 days in the experiment. Data are presented as average ± SEM. Flies showing an FFT value <0.01 were counted as arrhythmic (AR). For the rhythmic flies (including those that showed shifts of activity in DD), circadian period was determined by chi-square periodogram analysis.

### Old flies have weaker rhythms in the presence of light–dark cycles and lack the robust circadian morning peak of activity

We reported previously that rest–activity rhythms become weaker with age (Koh *et al.*, 2006). In the current study, despite the changes described above, the circadian rhythm was generally found to be more robust, at least for the first few days in DD. Because the previous experiments monitored activity in the presence of light–dark cycles, whereas here we assayed DD behavior so as to focus on circadian regulation, it seemed likely that the differences arose from the different environmental conditions. Indeed, when we compared the LD and DD behavior of old flies, we found that the overall rhythm is much weaker in LD (Table 1). As noted below, this may be due, in part, to inhibitory effects of light on aspects of oscillator function. Alternatively, or in addition, it could reflect circadian-independent influences of age on sleep (see Discussion).

**Fig. 2:**
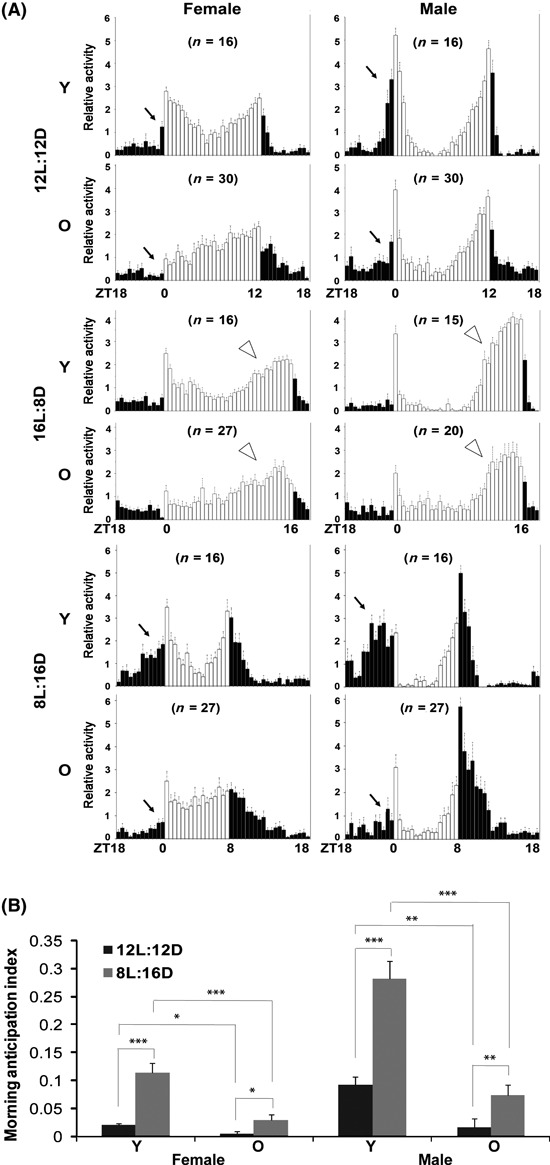
The circadian morning peak of activity is weakened in old flies. (A) In a 12:12 h light–dark cycle (12 L:12 D), young (10 days, Y) flies have robust bimodal activity peaks, but old flies (53 days, O) have a reduced morning peak of activity and increased early night activity. Under a 16:8 h light–dark cycle (16 L:8 D), young flies display a dampening of the morning peak similar to that seen in old flies. However, a strong evening peak is present in both young and old flies. Under 8:16 h (8 L:16 D) short photoperiod conditions, young flies adapt with stronger bimodal activity peaks and enhanced morning anticipatory activity, while old flies still have less morning anticipatory activity. However, this short photoperiod consolidates activity to the early evening in the old flies. Raw activity numbers were normalized to the mean of each group and plotted in 30-min bins. Black arrows point to morning anticipatory activity, and white arrow heads point to evening peak activity. (B) Quantification of morning anticipation under different photoperiod conditions. Morning anticipation index was defined as increased activity before lights-on (anticipatory activity minus activity for the same length of time prior to anticipation) relative to activity over the entire 24 h day. In 12 L:12 D cycles, morning anticipation starts 30 min before lights-on in females and 1 h in males. In 8 L:16 D cycles, morning anticipation starts 3 h before lights-on in females and 4 h in males. Data are presented as the average ± SEM. *P < 0.05, **P < 0.01, ***P < 0.001, by Student’s. Flies were monitored first in 12 L:12 D for 3 days and subsequently monitored under the different photoperiod conditions indicated. Activity records from the 3rd day of each LD cycle were used for analysis. Individuals that survived less than 7 days during the experiment were excluded from the analysis.

Under normal 12:12 h LD conditions, a two oscillator network drives a bimodal pattern of activity that has pronounced morning and evening peaks of activity (Nitabach & Taghert, 2008). When we compared the behavioral profiles of young and old flies (53 days) in 12:12 h LD cycles, we noticed that old flies display reduced morning anticipation and peak activity and increased activity in early evening (Fig. 2A). To more carefully evaluate the effect of age on morning and evening oscillators, we exposed flies to different photoperiods that tend to favor one oscillator over the other. Because light inhibits output from the morning oscillator (Picot *et al.*, 2007), evening oscillator activity dominates under a long summer-like photoperiod (Stoleru *et al.*, 2007), while the morning oscillator dominates in short photoperiods. In a long photoperiod (16: 8h LD), young flies have a reduced morning peak, and so look quite similar to old flies (Fig. 2A). On the other hand, both groups of flies, even the old ones, display a strong evening peak (Fig. 2A). To test whether a shorter photoperiod could enhance the morning peak of activity in old flies, we exposed flies to a 8:16 h LD cycle. Consistent with previous observations (Shafer *et al.*, 2004), it dramatically increased morning anticipatory activity in young flies, but less so in old flies (Fig. 2A,B). Instead, the short photoperiod consolidated activity in the early evening in the old flies (Fig. 2A). Together, these data suggest that either the function of or the output from the morning oscillator is weakened during aging.

### Molecular circadian oscillations are reduced in peripheral tissues during aging

**Fig. 3:**
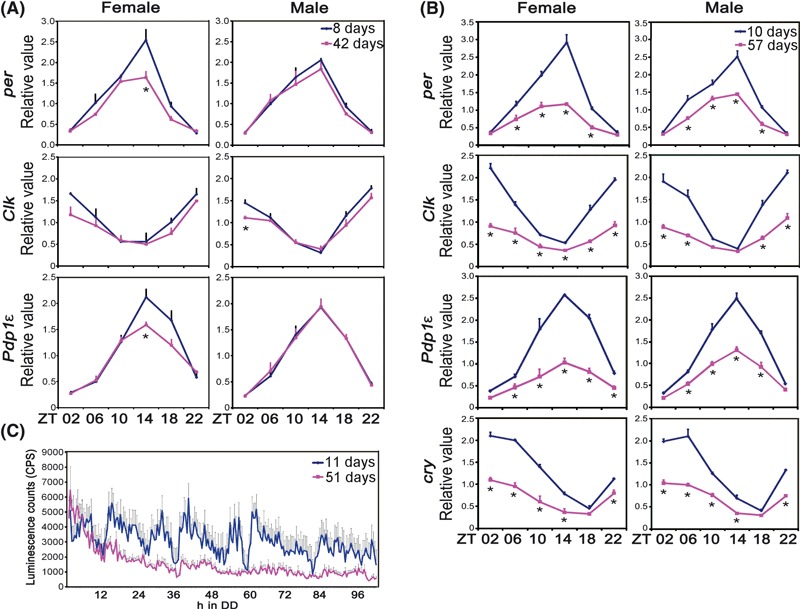
The molecular circadian clock is dampened in peripheral tissues of old flies. (A, B) Total mRNA extracted from fly heads (8 days and 42 days flies in panel A, 10 days and 57 days flies in panel B) at different times of day was analyzed by qPCR. Clock gene mRNA levels were normalized to that of *Actin* RNA. Data are averaged from three independent experiments and error bars represents standard error of mean. ZT denotes Zeitgeber time (where ZT0 is light on and ZT12 is light off in a 12:12 h light–dark schedule). The signal in adult heads is derived largely from the eye. As in the case of the behavior, dampening of molecular oscillations occurs earlier and is more pronounced in females. *P < 0.05, **P < 0.01, ***P < 0.001, by pair-wise Student’s *t*-test. (C) Whole-fly *per*-luciferase (BG-*luc*) reporter expression is dampened in aged flies. Male flies were loaded into a luminometer during the light–dark transition and luciferase activity was measured for 5 days in constant darkness. Data are shown as the average ± SEM of young flies (11 days, *n* = 20) or of aged flies (51 days, *n* = 18).

To determine the effects of aging on the molecular oscillator, we first examined the expression of circadian clock genes at the mRNA level in fly heads. The bulk of clock gene mRNA in the head is derived from the eye, which contains its own circadian clock (Plautz *et al.*, 1997) and thereby represents the activity of peripheral clocks. The amplitude of circadian clock gene expression under LD conditions is moderately reduced in middle age (42 days) (Fig. 3A), and is further reduced in older (57 days) flies (Fig. 3B). As in the case of the behavioral rhythms, molecular cycling of clock gene expression dampens earlier in female flies (Figs 3 and S2). This dampening of circadian gene expression is even more severe under free-running DD conditions (Fig. S2). Similarly, whole-fly expression of a *per*-luciferase reporter (BG-*luc*), which reflects activity of the *per* promoter in peripheral clocks throughout the fly body, is dramatically dampened in aged flies (Fig. 3C).

We also examined clock protein expression in adult heads through Western blot analysis and through immunofluorescence analysis of fly head sections. Peak expression of PER and TIMELESS (TIM) proteins in whole heads is reduced in old flies (58 days), although their circadian cycling persists under LD and DD conditions (Fig. S3). Similarly, PER protein oscillations in the compound eyes of old flies are blunted under LD conditions as shown in sections of fly heads (Fig. S4). These results indicate that the molecular circadian clocks in peripheral tissues are targets of the aging process.

### Robust circadian cycling of PER protein persists in the central clock neurons in aged flies

As noted, clock gene expression in peripheral tissues is reduced by age, and the cycling is dampened. We hypothesized that the circadian pacemaker in the central brain, which controls the circadian rest–activity rhythm, would also be affected by the aging process. To test this possibility, we examined the expression of the circadian clock protein PER in the brain clock neurons of aged flies.

Middle-aged (40 days) flies show robust circadian expression of PER on the first day of DD in three groups of clock neurons in the central brain: the small ventral lateral neurons (s-LN_v_s), the dorsal lateral neurons (LN_d_), and the dorsal neurons group1 (DN1) (Fig. S5). This observation correlates nicely with the circadian behavioral data because the majority of flies display rhythmic rest–activity in the first few days in constant darkness. We next asked whether disruption of the molecular circadian oscillator in the central brain underlies the instability of phase in older flies (60 days), such that females shift their phase of activity after 4–5 days in constant darkness. As shown in Fig. S6, PER expression in the s-LN_v_s is delayed in the 60-day-old females: it takes a longer time to accumulate in the late night/early morning, and so after 5 days in DD, peak expression of PER occurs at CT6 (CT0-subjective dawn and CT12-subjective dusk, assuming a 24 h cycle in DD). The peak at ∼CT6, rather than at ∼CT2, as seen in young flies (Fig. S5), is consistent with the approximately 0.8 h longer period seen for behavioral rhythms (given that five cycles have occurred in DD) (Table 1). On the other hand, robust cycling of PER persists in old flies (Fig. S6). Thus, the behavioral changes that occur in 56% of old female flies (i.e., shifting at DD4, unstable phase) do not seem to be caused by decreased amplitude of the molecular circadian clock or loss of synchrony between different clock neurons (Fig. S6).

**Fig. 4:**
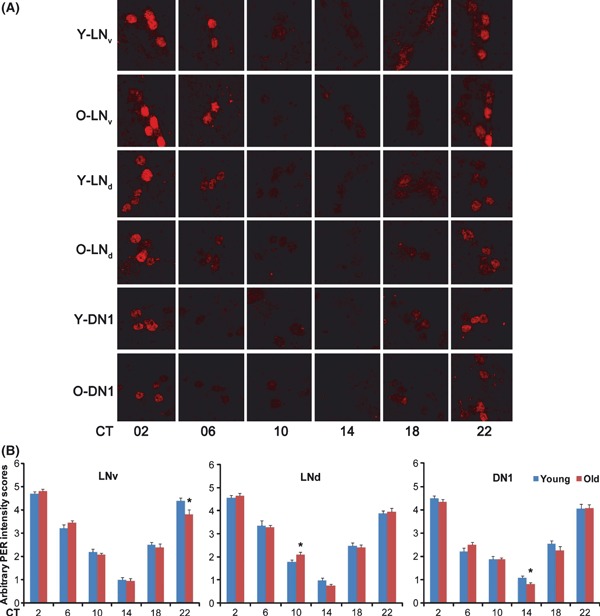
Robust PER cycling persists in terminal age, arrhythmic flies. (A) 80 days old (O) female flies were monitored for their circadian behavior and arrhythmic flies were examined for PER expression on day 3–4 in constant darkness, along with 8-day-old (Y) young female control flies. Both young and aged flies have robust and synchronized cycling of PER in three groups of circadian clock neurons shown. More than ten brains were examined for each time point and representative images are shown. (B) Arbitrary intensity scores for PER staining in clock neurons. PER intensity was objectively scored by eye (1 is the lowest and 5 is the highest). The average ± SEM of all hemispheres (*n* > 14) examined is shown. *0.01 < *P* < 0.05, by Student’s *t*-test.

To further test whether behavioral arrhythmicity at the terminal life stage is caused by defects in the molecular circadian clock, we first assayed circadian behavior of 80-day-old flies in DD and then selected arrhythmic flies for analysis of PER expression. To our surprise, terminal-age arrhythmic flies display robust circadian expression of PER, generally comparable to that in young flies, in all three groups of clock neurons (Fig. 4A,B). Importantly, the phase of the PER oscillation is well synchronized among these three groups of clock neurons (Figs 4 and S7).

These findings strongly indicate that compromised output from the central pacemaker, rather than deterioration of the molecular circadian clock, underlies the circadian abnormality in aging flies. Given the reduced morning peak of activity in old flies, it is possible that output from the morning oscillator, which consists predominantly of the small LN_v_s (Grima *et al.*, 2004; Stoleru *et al.*, 2004), is affected preferentially. PDF is a major secreted output of the central clock, specifically of the small and large LN_v_s. The dorsal projection of the small LN_v_s is thought to be particularly important for rest–activity rhythms and so we examined PDF-containing arborizations of this projection in old flies. We found that PDF intensity at the dorsal termini is slightly reduced in aged (82 days) flies (Fig. S8). However, it is not clear whether this reduction contributes to the behavioral phenotype (see Discussion).

### Coupled light–dark and temperature cycles improve rhythms in old flies

**Fig. 5:**
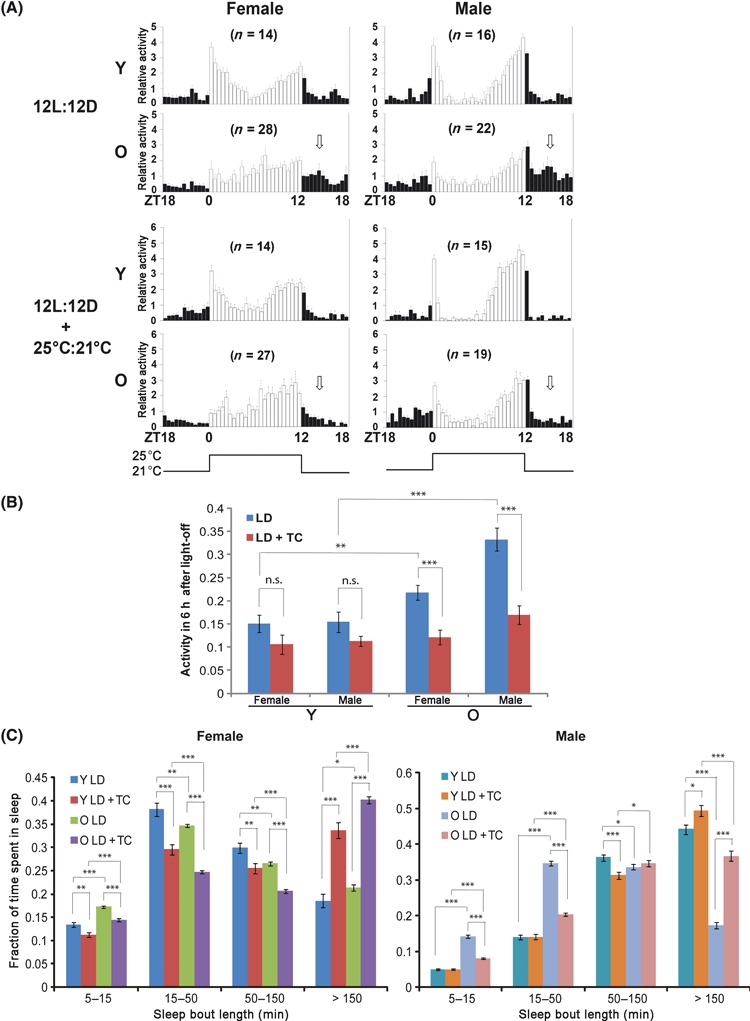
The rest–activity rhythm of old flies is enhanced when the light–dark cycle (LD) is coupled with a high–low temperature cycle. (A) Old flies have more consolidated daytime activity and less night activity (white arrows), and thus a stronger rest–activity cycle, when the 12 L:12 D cycle is coupled with a 25:21 °C temperature cycle. ‘Y’ denotes young flies (9 days) and ‘O’ denotes old flies (57 days). Raw activity numbers were normalized to the mean of each group and plotted in 30-min bins. (B) Quantification of early night activity under different entrainment conditions. Early night activity was quantified as ratio of activity during the 6 h after lights-off over that of the entire 24 h day and presented as average ± SEM. (C) Raw data from the above experiments were further analyzed for sleep consolidation. LD combined with a temperature cycle (TC) improves sleep consolidation, such that old female and male flies show a larger fraction of long sleep bouts (>150 min). *P < 0.05, **P < 0.01, ***P < 0.001, n.s. (no significant difference) by Student’s *t*-test. Activity records from the 3rd day of the LD cycle or combined LD and temperature cycle were used for analysis. Individuals that survived < 7 days during the experiment were excluded from the analysis.

We next sought to identify conditions that could improve rhythms in old flies. Because rhythmic behavior in environmental cycles reflects not only circadian regulation, but also direct effects of the entraining stimulus on activity (an effect known as masking), we reasoned that it might be possible to enhance these rhythms by altering the entraining stimulus. As noted above, altering the amount of light with a short photoperiod consolidated activity to evening hours in old flies (Fig. 2A). Besides the light–dark cycle, temperature is a potent environmental factor that entrains the circadian clock. To test whether two entraining stimuli could strengthen the rest activity cycle in old flies, we coupled the LD cycle with a temperature cycle such that the light phase was accompanied by a temperature of 25 °C and the dark phase with 21 °C. We found that superimposition of these two cycles consolidated activity to daytime, thereby considerably decreasing early night activity in old flies (Fig. 5A,B). Sleep was also improved in old flies under these conditions, such that more of the sleep occurred in long bouts (>150 min) (Fig. 5C). Such a shift toward long bouts, with a corresponding decrease in short bouts, is a reflection of sleep consolidation. These data suggest that stronger environmental entraining stimuli improve the strength of behavioral cycles in old flies, at least partially through consolidating sleep.

### Reducing PKA activity mitigates age-related circadian behavioral defects

To enhance circadian rhythms in free-running conditions, we sought to manipulate candidate molecules known to function downstream of the clock. The cAMP-PKA signaling pathway is implicated in circadian function, in particular in the output pathway (Levine *et al.*, 1994; Majercak *et al.*, 1997), and has also emerged as an attractive anti-aging target (Enns & Ladiges, 2010). For instance, in *Drosophila*, age-related memory impairments can be suppressed by reducing PKA activity (Yamazaki *et al.*, 2007). Thus, we considered the possibility that changes in PKA signaling account for the circadian aging phenotype.

**Fig. 6:**
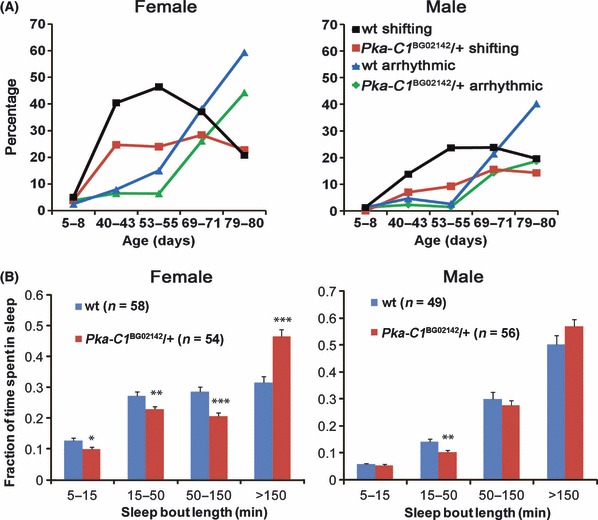
Reduced protein kinase A (PKA) expression improves circadian behavioral rhythms and sleep consolidation in aged flies. (A) Compared to sibling controls (wt), percentage of flies showing unstable (shifting) and arrhythmic behavior is reduced in *Pka-C1*^BG02142^/+ mutants. (B) *Pka-C1*^BG02142^/+ mutants spend less time in shorter sleep episodes than sibling controls. This effect is more prominent in females. Flies aged at 55 days were monitored under LD conditions and data were collected in 1-min bins. Data are presented as mean ± SEM. *P < 0.05, **P < 0.01, ***P < 0.001, by Student’s *t*-test.

The phenotype of old flies is generally consistent with an increase, rather than a decrease, in PKA signaling given that *Pka-C1* mutants have a shorter circadian period (Majercak *et al.*, 1997). To test this, we first asked whether elevation of PKA signaling leads to aging-like circadian behaviors. As shown in Fig. S9A, induced overexpression of PKA catalytic subunit in the adult nervous system causes arrhythmia. To test whether reducing PKA activity could rescue age-associated circadian defects, we examined aged flies that have reduced expression of *Pka-C1* (30% reduction in mRNA levels in young heterozygous flies as measured by quantitative real-time PCR). Indeed, these aged mutant flies have stronger circadian behavioral rhythms compared to sibling controls under constant darkness conditions (Fig. 6A, Table 2). The number of flies displaying an unstable phase is reduced, and the overall rhythm strength is enhanced. However, these flies still have a long circadian period, similar to that of sibling controls (Table 2). Another allele of *Pka-C1* mutant (10% reduction in mRNA levels in young heterozygous flies) also partially rescues the arrhythmic phenotype of 60-day-old flies (Fig. S9B). Because old flies have a robust molecular oscillator in the central brain, it is possible that reducing PKA activity restores circadian output function. Alternatively, reducing PKA activity may consolidate sleep, which in turn improves the strength of the sleep–wake cycle. In support of the latter, we previously found that increased PKA activity fragments sleep (Joiner *et al.*, 2006). Indeed, when flies were monitored under LD conditions typically used for sleep assays, aged female *Pka-C1* mutants displayed improved sleep consolidation such that long sleep bouts were increased, and the total number of bouts per day was decreased relative to age-matched sibling controls. Male *Pka-C1* mutants also showed a small improvement in sleep consolidation (Figs 6B and S10).

**Table 2:** Reduced *Pka-C1* expression partially rescues circadian behavioral rhythms

	Age (days)	5–8	40	53–55	69–71	79–80
Female						
wt	*n*	80	89	140	97	101
	Period (h)	24.07 ± 0.03	24.72 ± 0.06	25.08 ± 0.04	24.76 ± 0.06	24.89 ± 0.12
	FFT	0.055 ± 0.003	0.048 ± 0.003	0.054 ± 0.002	0.037 ± 0.003	0.045 ± 0.004
	AR (%)	2.5	7.9	15.0	38.1	59.4
	Shifting (%)	5.0	40.5	46.4	37.1	20.8
*Pka-C1*^BG^/+	*n*	79	93	125	88	79
	Period (h)	23.97 ± 0.03	24.49 ± 0.07	25.02 ± 0.04	24.75 ± 0.08	24.61 ± 0.09
	FFT	0.041 ± 0.003	0.044 ± 0.002	0.063 ± 0.003	0.040 ± 0.003	0.035 ± 0.004
	AR (%)	3.8	6.5	6.4	26.1	44.3
	Shifting (%)	3.8	24.7	24.0	28.4	22.8
Male						
wt	*n*	80	87	114	84	97
	Period (h)	23.77 ± 0.02	24.52 ± 0.05	24.67 ± 0.04	24.85 ± 0.11	25.24 ± 0.10
	FFT	0.105 ± 0.005	0.061 ± 0.003	0.059 ± 0.002	0.052 ± 0.003	0.042 ± 0.003
	AR (%)	1.3	4.6	2.6	21.4	40.2
	Shifting (%)	1.3	13.8	23.7	23.8	19.6
*Pka-C1*^BG^/+	*n*	79	86	130	77	91
	Period (h)	23.79 ± 0.02	24.22 ± 0.06	24.88 ± 0.05	24.92 ± 0.09	24.80 ± 0.10
	FFT	0.103 ± 0.005	0.052 ± 0.002	0.063 ± 0.003	0.051 ± 0.004	0.057 ± 0.003
	AR (%)	1.3	2.3	1.5	14.3	18.7
	Shifting (%)	0	7.0	9.2	15.6	14.3

Siblings from the seven times outcrossed *Pka-C1*^BG02142^ strain were used as wild-type control for the mutants. Data are presented as average ± SEM. Flies with an FFT value <0.01 were counted as arrhythmic (AR). For the rhythmic flies, circadian period was determined by chi-square periodogram.

## Discussion

### Circadian rest–activity behavior degenerates with age

Deterioration of circadian rhythms is a prominent symptom of aging (Turek *et al.*, 1995; Hofman & Swaab, 2006). We report here that the endogenously driven rest–activity rhythm degenerates with age in flies. In addition to reduced rhythmicity and a longer circadian period, some aged flies show an unstable phase, which manifests as an expansion and contraction of activity duration: a daily shortening of the duration of activity (alpha) is followed by expanded activity (expansion of alpha) on days 4–6. In some aged flies, the gradual contraction of alpha is not obvious, but they still have an expansion of alpha on days 4–6. This contraction–expansion of alpha repeats every 4–5 days in aged females and every 5–6 days in aged males (Figs 1A and S1). Flies also exhibit gender dimorphism of age-associated alteration of circadian behavioral rhythms: at any given age, a greater percentage of female flies shows defects in circadian rhythms than males (Fig. 1B, Table 1), although their lifespans are comparable (Koh *et al.*, 2008).

Because the effect on circadian period coincides with rhythm deterioration, it is tempting to speculate that period lengthening is an underlying cause of the aged-associated weakening of circadian rhythms. However, we were unable to correlate these two parameters in individual flies. In addition, the circadian period does not get any longer after middle age in females, suggesting that it is most likely an independent target of the aging process. Another *Drosophila* species (*D. nasuta*) also shows period lengthening and rhythm degeneration with age (Joshi *et al.*, 1999), and the free-running rhythm period also lengthens in old mice (Mayeda *et al.*, 1997). In humans and some other mammalian species, circadian period shortens with age (Pittendrigh & Daan, 1974; Witting *et al.*, 1994; Monk, 2005; Aujard *et al.*, 2006), but this may not underlie sleep–wake disturbances reported in humans (Duffy & Czeisler, 2002).

### Peripheral circadian clocks are targets of the aging process

The amplitude of circadian clock gene expression dampens in heads of old flies (Figs 3A,B, and S2–S3). This is indicative of reduced amplitude in peripheral clocks because the major molecular clock signal in heads comes from the compound eye (Plautz *et al.*, 1997). Indeed, we found that PER oscillations in the photoreceptor cells are dampened (Fig. S4). Interestingly, the cycling of clock proteins PER and TIM is not as severely dampened as is the mRNA cycling in 58-day-old flies (Figs 3 and S3). This is particularly true under LD conditions, where robust circadian expression of the clock proteins persists, albeit with reduced peak expression levels. It appears that aging affects circadian clock gene expression mainly at the transcriptional level. Consistent with this, *per* promoter activity is reduced in peripheral clocks throughout the fly body (Fig. 3C). Altered clock gene transcript levels have been reported in aged vertebrate species, and in *Drosophila: Per2* and *Bmal1* mRNA expression is reduced in peripheral tissues of aged mice (Kunieda *et al.*, 2006); circadian oscillations of a *Per1*-*luc* reporter are abrogated in some peripheral tissues but robust in other tissues (Yamazaki *et al.*, 2002); changes in *Bmal1* (but not *Clock*, *Per1*, and *Per3*) levels correlate with age in human peripheral blood cells (Ando *et al.*, 2010); age-dependent reduction in *Bmal1* and *Per1* (but not *Clock*) mRNA expression occurs in the zebrafish eye (Zhdanova *et al.*, 2008); and finally, *per* mRNA is reportedly dampened in the heads of old (50 days) flies (Krishnan *et al.*, 2009). Our finding that expression of all clock genes is dampened provides clear evidence that the circadian clock machinery, but not just the expression of specific clock genes in peripheral tissues, is targeted by the aging process.

### Robust molecular circadian oscillations persist in the central pacemaker during aging

The long circadian behavioral period in old flies correlates nicely with a slower pace of PER oscillation in the small LN_v_s (Fig. S6). However, the cyclic expression of PER is robust and synchronized among different groups of clock neurons in aged flies (Figs 4 and S6). This important finding is corroborated by some reports from mammalian studies: robust circadian expression of *Per1, Per2, Cry1* mRNA and a *Per1*-luciferase reporter persists in the SCN of old rats (Asai *et al.*, 2001; Yamazaki *et al.*, 2002), and PER2 rhythm is largely intact in middle-aged mice (Asai *et al.*, 2001; Yamazaki *et al.*, 2002). Thus, despite some reports of reduced circadian clock gene mRNA expression in the SCN of old animals (Weinert *et al.*, 2001; Kolker *et al.*, 2003), there is support for the idea that the molecular central clock is largely intact during the aging process.

### Aging weakens output from the morning oscillator

So what is affected by age in the central timekeeping mechanism? We suggest that apart from the change in the free-running period, age affects the output of the central clock, rather than the clock itself. An effect on output may be more evident under certain conditions. Indeed, we found that aged flies have weaker behavioral rhythms under normal 12 L:12 D schedules than in constant darkness (Table 1). Further examination of the LD profile reveals that aged flies have a dramatically reduced morning peak of activity, while evening activity is extended. Even under short photoperiods, when output from the morning oscillator is dramatically enhanced in young flies, old flies still display much less morning anticipatory activity, suggesting that output from the morning oscillator is weakened by age (Fig. 2). Typically, masking effects of light may help to consolidate sleep and these effects may become weaker with age. Some environmental manipulations (e.g., LD cycles together with temperature cycles) improved the strength of the rest–activity cycle, perhaps by providing stronger masking signals that enabled better consolidation of activity and sleep (Fig. 5).

A weakened morning oscillator may also account for the unstable behavioral rhythms in DD (see above). The morning oscillator reportedly sets the timing of the evening peak (Stoleru *et al.*, 2005). We propose that in young flies, strong output from the morning oscillator sets the evening peak to occur at a 180° angle; in aged flies, weakened output from the morning oscillator relaxes the phase restraint on the evening oscillator, thus allowing the evening activity peak to gradually advance into the territory of the morning oscillator. However, this advance is ultimately pushed back by the weakened morning oscillator, typically on the 4–5th day in DD for females and 5–6th day for males. This contraction–expansion of alpha manifests in many different ways (Fig. S1), which is typical of age-related physiological changes.

An effect on morning and evening peaks is reminiscent of the action of the PDF neuropeptide: mutants lacking PDF or its receptor display a weakened morning anticipatory activity peak and an earlier evening activity peak in LD (Lear *et al.*, 2009; Shafer & Taghert, 2009). We observed a small reduction in PDF intensity at the terminus of the dorsal projection from the small LN_v_s. However, we do not believe this change of PDF intensity is responsible for the phenotype we observe in aged flies. For one, old flies display a longer period, while *Pdf* mutants have a short period (Yoshii *et al.*, 2009); also, they do not display the typical advance of evening activity seen in *Pdf* mutants. It is possible that, in addition to PDF, the morning oscillator employs other factor(s) to regulate morning and evening activity (Shafer & Taghert, 2009).

### Reducing PKA improves circadian behavioral rhythms in aged flies

Normal aging is often accompanied by impaired memory. Interestingly, increasing PKA activity impairs, while decreasing PKA activity ameliorates, deficits in prefrontal cortex memory function in aged rats and monkeys (Ramos *et al.*, 2003). Similarly, reduction in PKA expression (by 40%) suppresses age-related memory impairment (AMI) in flies (Yamazaki *et al.*, 2007). We report here that a reduction in PKA partially rescues age-related circadian rhythm deterioration (Table 2, Figs 6A and S9B) although it does not rescue the long circadian period. Because PKA is implicated in clock output (Levine *et al.*, 1994; Majercak *et al.*, 1997), it is possible that the decrease in PKA down-regulates the output from the evening oscillator and thus counters the weakened output from the morning oscillator. Alternatively, or in addition, it may strengthen behavioral rhythms by consolidating sleep, as elevated PKA activity fragments sleep in young flies (Joiner *et al.*, 2006). Indeed, we show here that at least some of the rhythm-strengthening effects of reduced PKA are likely due to better consolidation of sleep (Figs 6B and S10).

Reduced PKA signaling reportedly extends lifespan in yeast (Lin *et al.*, 2000) and promotes healthy aging in mice. Because a 40% reduction in PKA activity does not affect lifespan in *Drosophila* (Yamazaki *et al.*, 2007), we do not expect that this *Pka-C1*^BG02142^ allele, which reduces *Pka-C1* expression approximately 30%, extends lifespan. We note that our data do not necessarily imply that PKA activity is up-regulated in aged flies. Reducing PKA expression may correct the regulation of a PKA target that is up- or down-regulated by PKA-independent pathways during aging. Nonetheless, our data demonstrate that reducing PKA expression can rescue some aspects of age-related circadian behavioral defects and thus provide a promising target for therapeutic interventions aimed at ameliorating age-related circadian disorders.

## Experimental procedures

### Fly strains and aging procedure

All fly stocks were maintained on standard molasses-cornmeal-yeast food. An isogenic *w*^1118^ (iso31) strain was used as wild-type in this study (Koh *et al.*, 2008). For the aging experiment, iso31 flies were collected within 1–2 days after eclosion from density-controlled cultures and maintained in a vial with 15 pairs of females and males. Flies were kept in 12:12 h light–dark cycles at 25 °C and flipped into fresh vials every 4 days during the light phase. Progenies of approximately 30 to 40-day-old flies aged in this fashion were used as young controls. *Pka-C1*^BG02142^ (P element insertion in the 5′ UTR) and *Pka-C1*^EY08221^ (P element insertion in the promoter) mutations were outcrossed into the iso31 background for 5–7 generations to generate mutant and sibling control progenies.

### Circadian behavioral and sleep assays

To assess the circadian locomotor activity rhythm, light–dark cycle-entrained young and old flies were loaded in glass tubes containing 5% sucrose and 2% agar food. Locomotor activities were monitored for more than 7 days using the *Drosophila* Activity Monitoring System (Trikinetics, Waltham, MA, USA) in a 12:12 h light–dark (LD), constant darkness (DD) or varying photoperiod at 25 °C (except the temperature cycle experiment where 25 °C is coupled to light phase and 21 °C is coupled to dark phase). Activity counts were collected in 5-min bins and analyzed using ClockLab (Actimetrics, Wilmette, IL, USA). Rhythmicity is determined by chi-square periodogram analysis for flies that have activity records for more than 5 days. Flies with a FFT (fast Fourier transform) value lower than 0.01 were counted as arrhythmic. For sleep analysis, flies were monitored under LD condition, and locomotor activity data were collected in 1-min bins. Raw data from first day were discarded. Consecutive immobility of >5 min was counted as a sleep bout.

### Whole-fly luciferase reporter activity assay

Female *per-luciferase* transgenic flies (BG-*luc*) were crossed to iso31 male flies and progenies were collected and aged as described above. Young and old male flies were loaded into an opaque 96-well plate that contains 500 μm firefly luciferin (Biosynth AG, Rietlistr, Switzerland) mixed in food with 5% sucrose and 2% agar. The plate was then loaded into a Topcount NXT luminometer (PerkinElmer, Waltham, MA, USA) at the light–dark transition of the LD cycle and monitored in constant darkness over 5 days at 25 °C. Each well was read twice in a 30-min interval. Raw data were imported and analyzed in Excel.

### Quantitative real-time PCR and Western blot analysis

Young and aged flies were collected on dry ice at indicated time points in LD or DD. Total RNA was isolated using Trizol isolation system (Invitrogen, Carlbad, CA, USA), and cDNAs were synthesized by using a high-capacity cDNA Archive kit (Applied Biosystems, Foster City, CA, USA). Quantitative real-time PCR was performed in an ABI prism 7100 using a SYBR Green kit (Applied Biosystems). For Western blot analysis, flies were collected at indicated time points in LD or DD and fly heads were separated for protein extraction using standard cell lysis protocol. After SDS–PAGE, proteins were transferred onto nitrocellulose membrane and processed for antibody incubation. Primary antibodies Rabbit anti-PER (PA1139) and Rat anti-TIM (UPR41) were used at 1:1000 dilution. Following enhanced chemiluminescence, images were taken in a Kodak image station or exposed to a film, and images were processed using Adobe Photoshop (Adobe Systems Inc., San Jose, CA, USA).

### Immunohistochemistry analysis of whole-mount brain and frozen head sections

For whole-mount brain immunohistochemistry, young and aged flies were collected for brain dissection at indicated circadian times. Brains were fixed with 4% para-formaldehyde (PFA) in PBS (pH 7.4) for 45 min at room temperature. After three 15 min washes with 0.3% Triton X-100 in PBS, samples were incubated with 5% normal donkey serum in PBS-Triton X-100. Brains were then incubated with primary antibodies rat anti-PER (UPR34, 1:1000 dilution) and rabbit anti-PDF (HH74, 1:800 dilution) or mouse anti-PDF (C7; Developmental Studies Hybridoma Bank, 1:1000 dilution) overnight at 4 °C with gentle shaking. After three 20 min washes, brains were incubated with secondary antibodies donkey Cy3 anti-rat and FITC anti-rabbit or mouse (Jackson ImmunoResearch Laboratories, West Grove, PA, USA) for 2 h at room temperature, followed by extensive washes. Samples were mounted onto a slide with Vectashield mounting medium (Vector Laboratories, Burlingame, CA, USA) and imaged using a TCS SP5 confocal microscope (Leica Camera AG, Solms, Germany), with the same settings for all samples. Nine to twelve fly brains were examined for each time point. PER staining intensity was objectively scored by eye. At CT14, a few dorsal neurons near the vicinity of PDF projection that showed opposite phase of cycling to other clock neurons were excluded from scoring. Net pixel intensity of PDF staining at the terminus of the dorsal projection from the ventral lateral neurons was measured using a Kodak Molecular Imaging quantification program (Carestream Health Inc., Rochester, NY, USA).

For immunohistochemistry analysis of cryostat sections, 8–12 heads of young or old flies were collected at indicated Zeitgeber times. The proboscises were removed and the heads were fixed in 2% PFA in PBS for 90 min at 4 °C with gentle shaking. After three 15 min washes with PBS, samples were infiltrated with 12% sucrose in PBS overnight at 4 °C. The heads were then removed from the solution and embedded with a drop of O.C.T. TissueTek compound (Sakura Finetek, Torrance, CA, USA). The frozen samples were sectioned at 15 μm by using cryostat (Leica CM3050; Wetzlar, Germany), and sections were mounted onto Plus slides and kept at −20 °C. Slides were warmed up and fixed again with 0.5% PFA in PBS for 60 min at room temperature, followed by three quick washes. Slides were then incubated with 5% normal donkey serum in PBS-0.3% Triton X-100 for 2 h at room temperature and then incubated with primary rat anti-PER antibody (UPR34, 1:1000 dilution) overnight at 4 °C. After three 5 min washes, slides were incubated with secondary donkey Cy3 anti-rat antibody (Jackson ImmunoResearch Laboratories) for 2 h at room temperature, followed by four 5 min washes. Slides were mounted with Vectashield mounting medium (Vector Laboratories) and imaged using the TCS SP5 confocal microscope (Leica Camera AG, Solms, Germany).

## Supporting information

**Fig. S1** Aged flies display long period and unstable circadian behavior with varying degrees of expressivity.

**Fig. S2** Aged flies have dramatically reduced amplitude of circadian gene expression in constant darkness.

**Fig. S3** Circadian clock protein expression is dampened in peripheral tissues of old flies.

**Fig. S4** Aged flies have dampened amplitude of PER oscillation in the photoreceptor cells in the compound eyes.

**Fig. S5** Robust cycling of the circadian clock protein PER persists in middle-aged flies.

**Fig. S6** Molecular cycling of PER protein persists but delays in circadian clock neurons of aged flies.

**Fig. S7** The number of PER positive neurons is similar between young and terminal-age flies.

**Fig. S8** PDF expression in aged flies.

**Fig. S9** Increasing PKA signaling in young fliescauses arrhythmia, while a *Pka-C1* mutant allele partially rescues the arrhythmic phenotype of old flies.

**Fig. S10** Reducing PKA expression improves sleep maintenance in aged flies.

As a service to our authors and readers, this journal provides supporting information supplied by the authors. Such materials are peer-reviewed and may be re-organized for online delivery, but are not copy-edited or typeset. Technical support issues arising from supporting information (other than missing files) should be addressed to the authors.
