# Supplementary material for: Old flies have a robust central oscillator but weaker behavioral rhythms that can be improved by genetic and environmental manipulations
Source: Aging Cell. 2012 Jun;11(3):428–38. doi: 10.1111/j.1474-9726.2012.00800.x (PMC3353743; doi:10.1111/j.1474-9726.2012.00800.x)
Supplement: Supplementary file 1 [file acel0011-0428-SD1.doc]

**Old flies have a robust central oscillator but weaker behavioral rhythms that can be improved by genetic and environmental manipulations**

Wenyu Luo^1^, Wen-Feng Chen^2^, Zhifeng Yue^2^, Dechun Chen^3^, Mallory Sowcik^2^, Amita Sehgal^2,3*^ and Xiangzhong Zheng^3*^

1) Cell and Molecular Biology Program, 2) Howard Hughes Medical Institute, 3) Department of Neuroscience, University of Pennsylvania Perelman School of Medicine, Philadelphia, PA 19104

**Supplemental Figures**

**
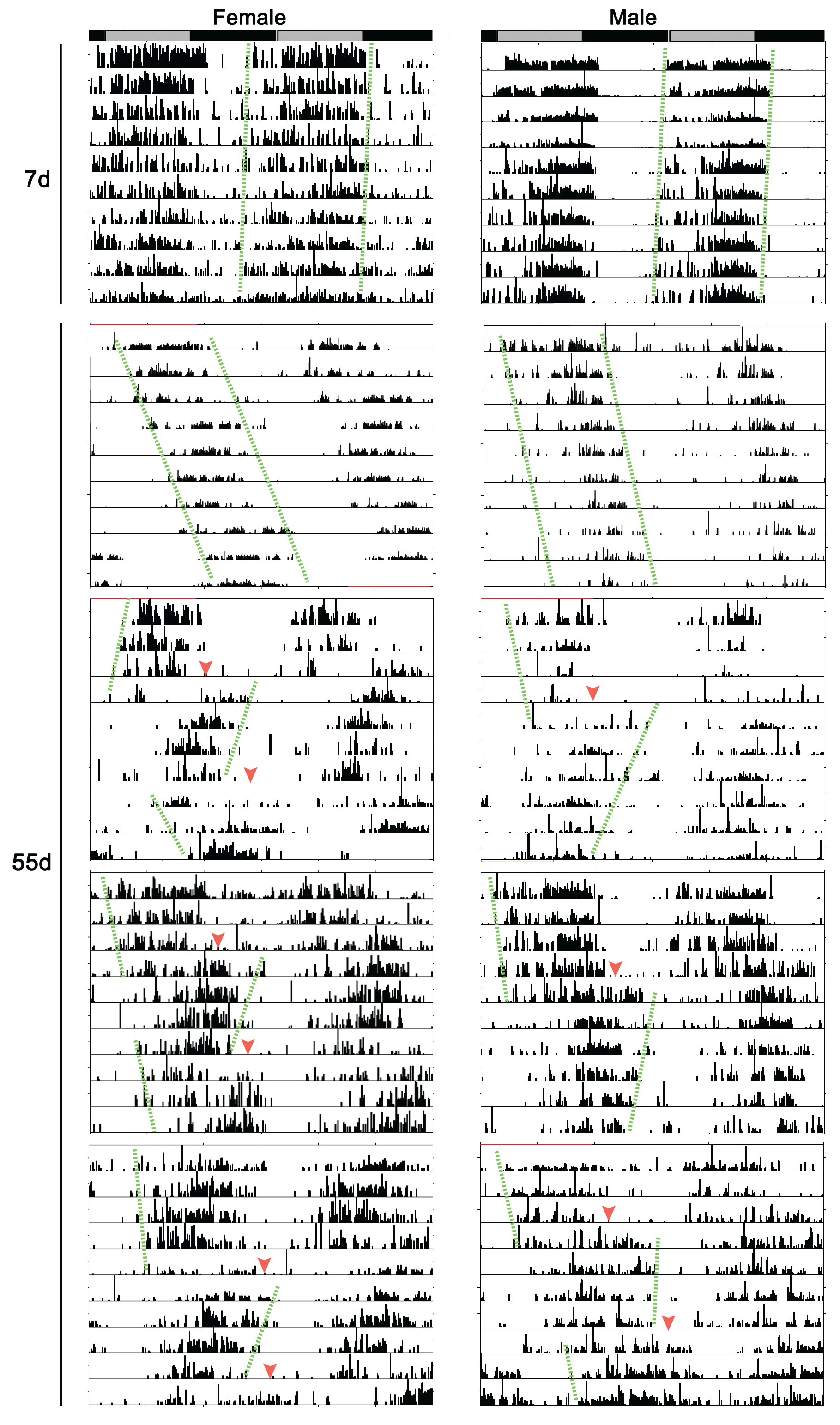
**

Figure S1. Aged flies display long period and unstable circadian behavior with varying degrees of expressivity. Most young (7d) flies have stable rhythms and a period closes to 24h. In contrast, most aged (55d) flies have long period and some aged flies show shifting of activity phase in constant darkness (also see Figure 1 and Table 1 in the main text for percentages). Dashed lines indicate segments with overt periods, and arrowheads indicate shifts in activity offsets. Some of the aged flies show a pronounced contraction of activity in the first few days, and then display a phase shift or an expansion of activity on days 4-6 of DD, followed by contraction of activity again. Female flies show more pronounced changes than male flies. The gray bar indicates subjective day, and black bar indicates subjective night.


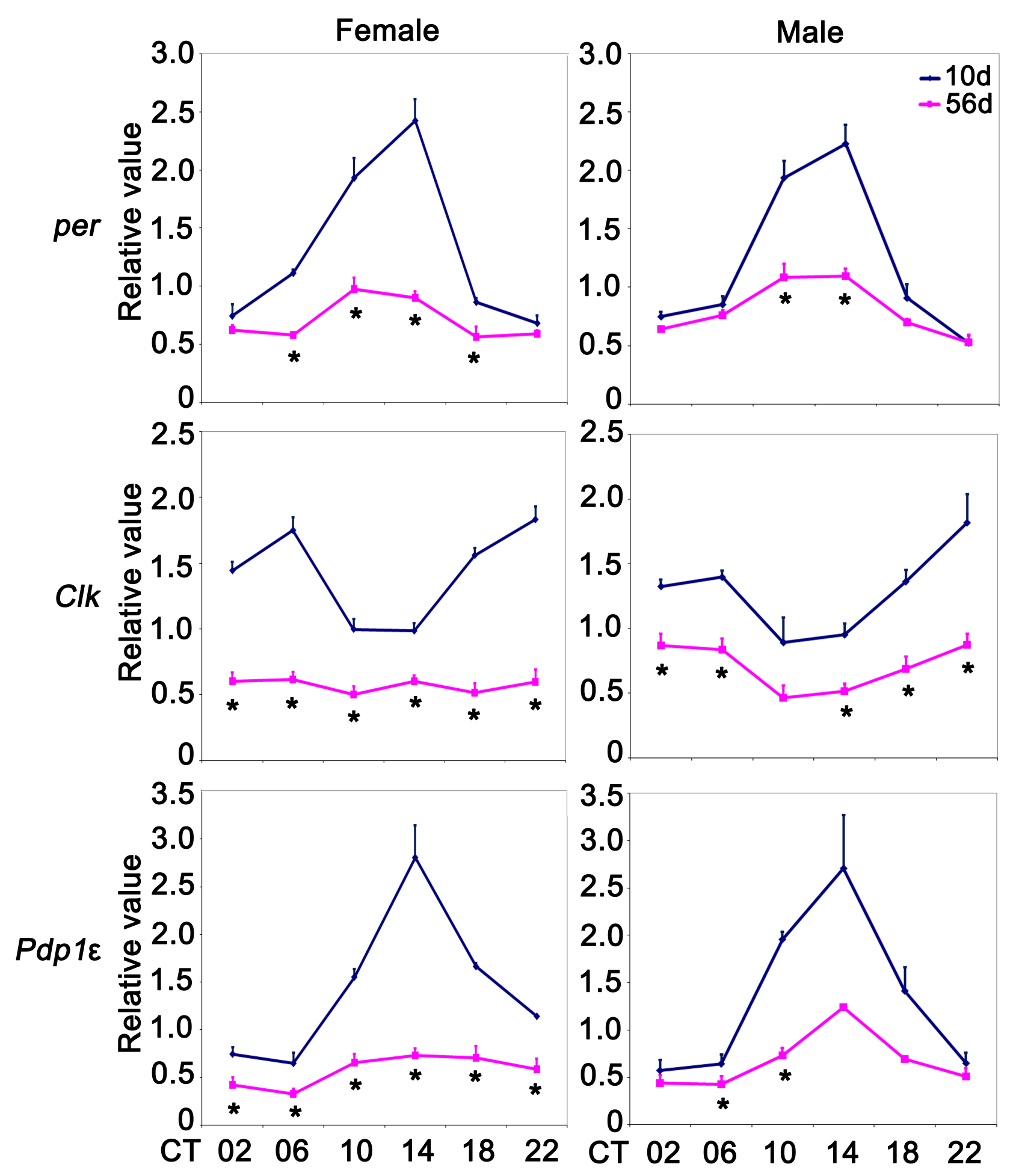


Figure S2. Aged flies have dramatically reduced amplitude of circadian gene expression in constant darkness. The amplitude of circadian mRNA expression of three clock genes (*per, Clk, Pdp1ε*) in 56-day-old flies is compared to that in 10-day-old young controls. Circadian clock gene mRNA expression is normalized to that of *Actin*. Data are presented as the average of 3 independent experiments and error bars denote standard error of the mean. *P<0.05, by pair-wise Student’s T-test.


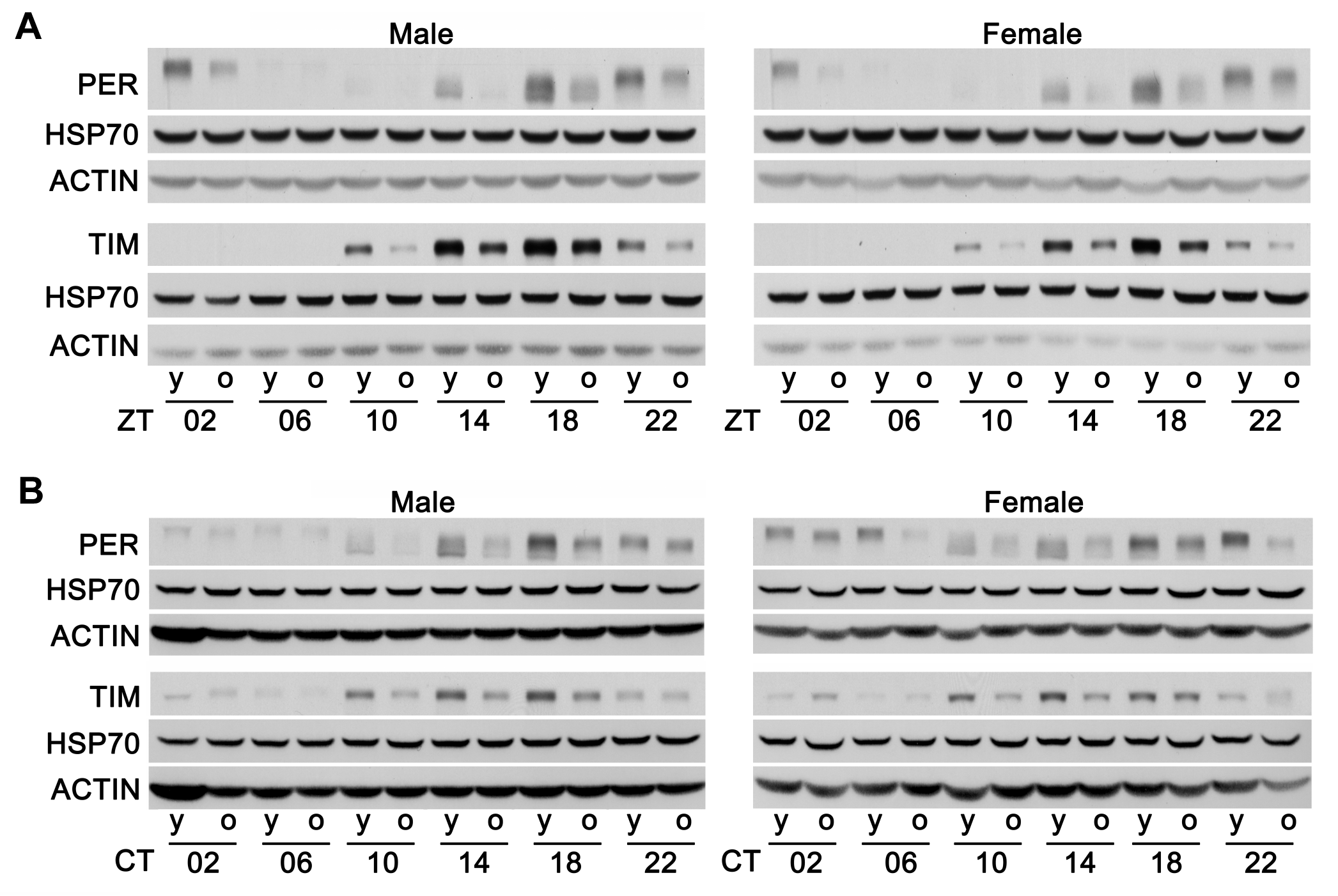


Figure S3. Circadian clock protein expression is dampened in peripheral tissues of old flies. Flies were entrained to 12h:12h light:dark cycles and whole heads were collected at indicated time points for Western blot analysis. Since most of the clock protein signal in adult heads comes from the compound eye, this assay mainly reports the state of peripheral circadian clocks. PER and TIM expression was assayed in LD (A) and DD (B). Compared to young flies (y, 8-day-old), aged flies (o, 58-day-old) show reduced peak expression of circadian clock proteins PER and TIM in light:dark (LD) cycles and circadian expression is further dampened in constant darkness (DD). Housekeeping proteins HSP70 and ACTIN were used as loading controls. Similar results were obtained from three independent experiments.


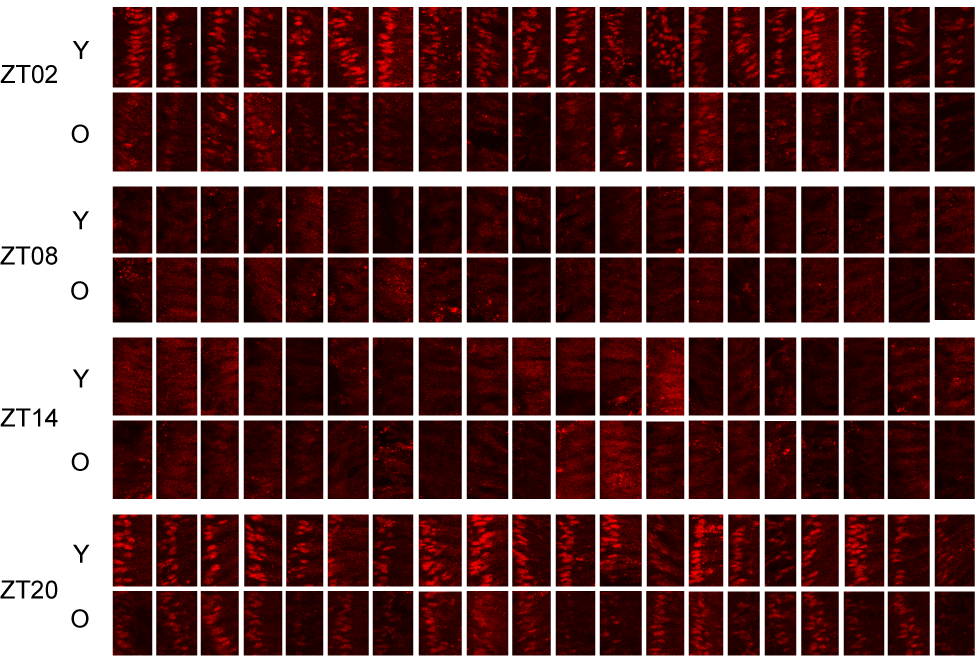


Figure S4. Aged flies have dampened amplitude of PER oscillation in the photoreceptor cells in the compound eyes. 57 days old (O) female fly head sections were examined for PER expression at indicated Zeitgeber times (ZT) in 12h:12h light:dark cycles, along with 8 days old young (Y) female control. Old flies have lower peak levels of PER (ZT02 and ZT20) in the photoreceptor cells as shown in multiple images. 8-12 fly heads were used for cryostat head sections at each time point.


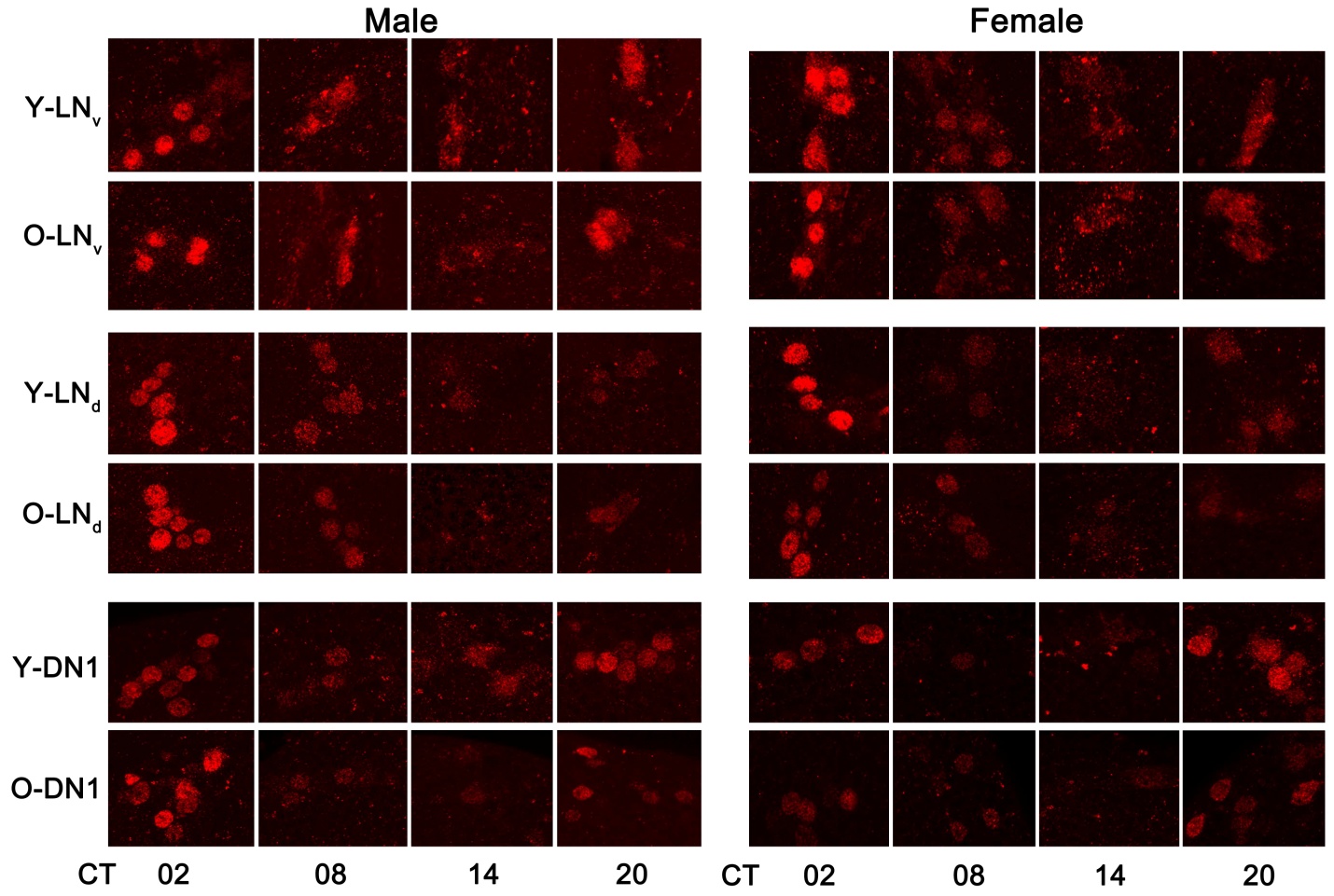


Figure S5. Robust cycling of the circadian clock protein PER persists in middle-aged flies. Flies were entrained to 12h:12h light:dark cycles and brains were dissected at indicated circadian time points (CT) on the first day of constant darkness. Both young (Y, 8-day-old) and middle-aged (O, 40-day-old) flies display robust cyclic expression of PER in all three groups of clock neurons. LN_v_ denotes small lateral neurons; LN_d_ denotes dorsal lateral neurons; DN1 denotes dorsal neuron group 1. Based upon analysis of 10-12 brains for each time point, representative images are shown.


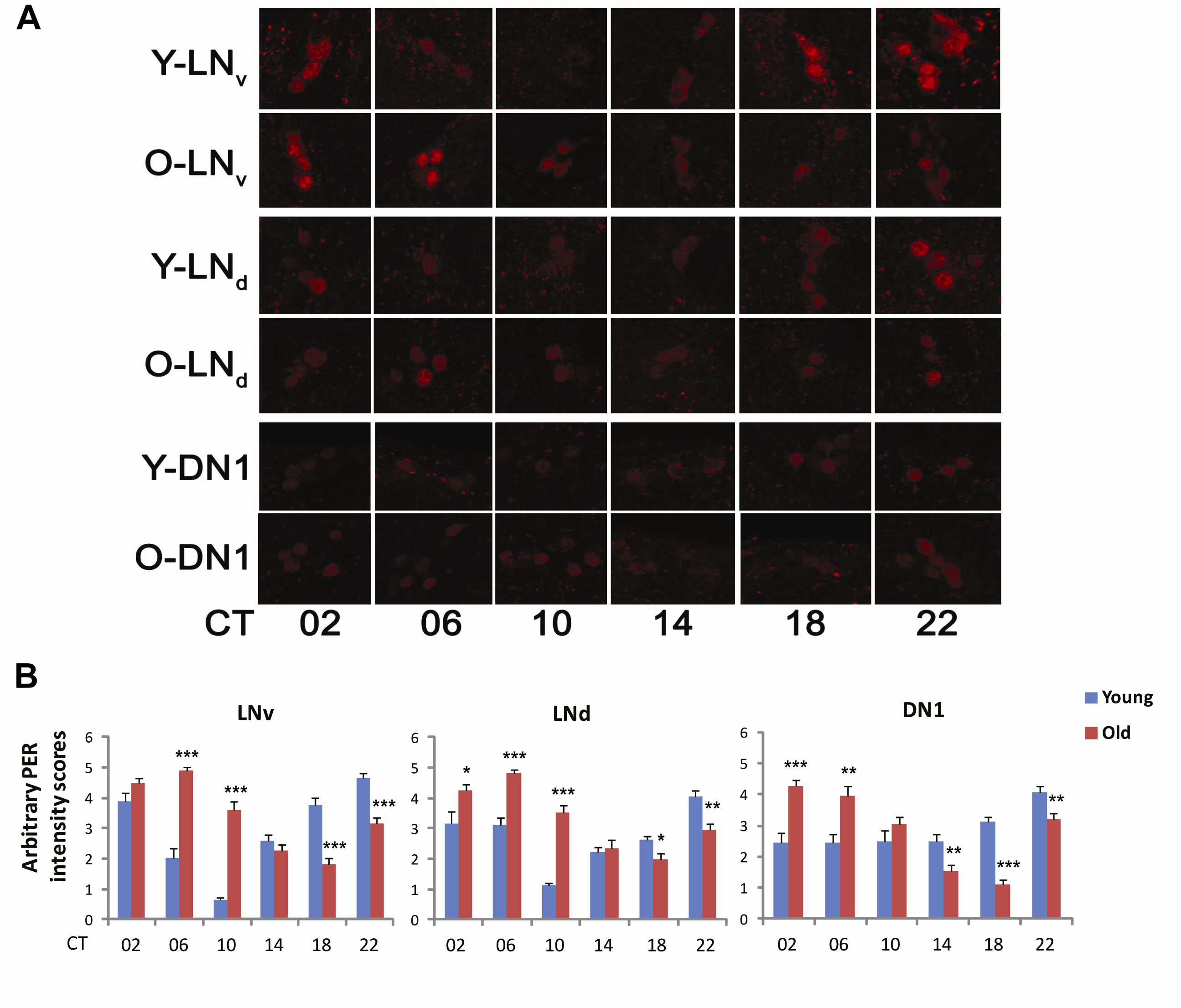
Figure S6. Molecular cycling of PER protein persists but delays in circadian clock neurons of aged flies. (A) 10-day-old (Y) and 60-day-old (O) female flies were collected at indicated circadian times (CT) on day 5-6 in constant darkness, and brains were dissected for immunofluoresence analysis of PER expression. For each time point, more than 10 brains were examined and representative images are shown. (B) Arbitrary intensity scores for PER staining in clock neurons. PER intensity was scored by eye (1 is the lowest and 5 is the highest). Data are presented as average ± SEM of all hemispheres examined (n>14). Both young and old female flies show robust circadian cycling of PER in the small ventral lateral neurons (LN_v_), the dorsal lateral neurons (LN_d_) and the dorsal neuron group 1 (DN1) in old flies. However, PER expression peaks at CT2-6 in all these groups in old female flies, which have longer circadian periods, rather than at CT18-22, as seen in young female flies. *P<0.05, **P<0.01, ***P<0.001, by Student’s T-test.


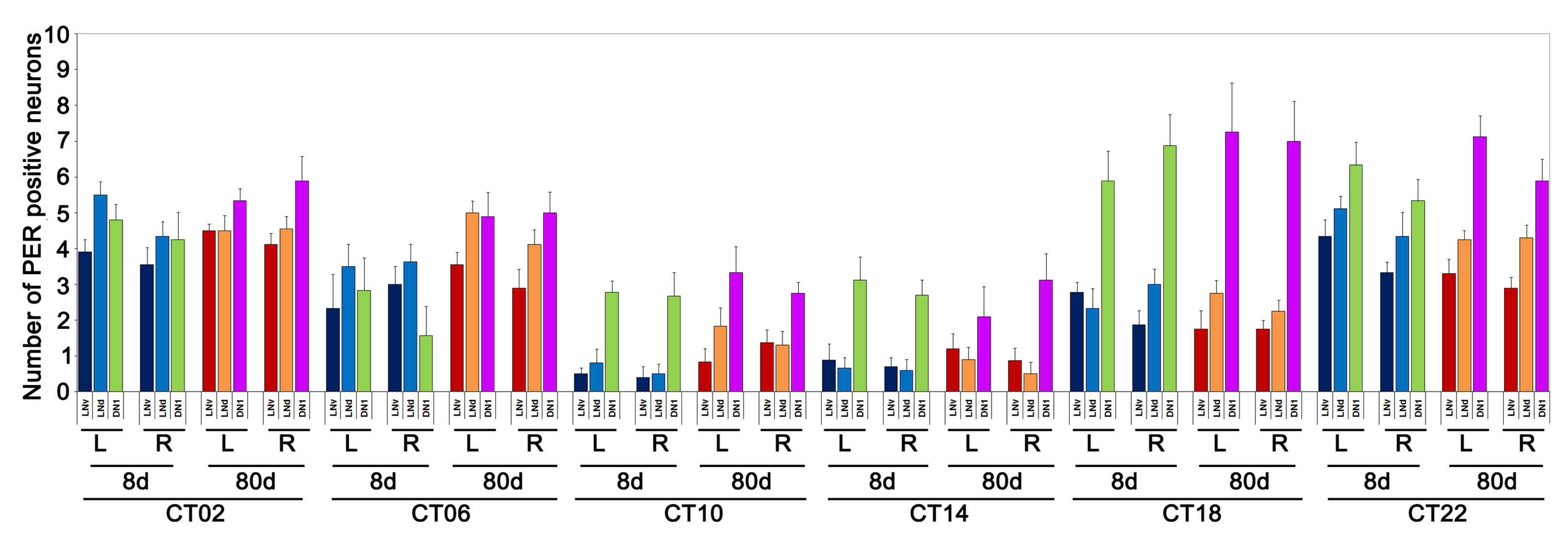


Figure S7. The number of PER positive neurons is similar between young and terminal-age flies. Circadian behavior was monitored for 3 days in constant darkness. Rhythmic young (8d) and arrhythmic old (80d) female flies were collected for brain dissection and immunohistochemistry for PER expression (also see Figure 4 in the main text). The number of PER positive neurons was counted for all three groups of clock neurons in each brain hemisphere: small ventral lateral neurons (LN_v_), dorsal lateral neurons (LN_d_) and dorsal neuron group1 (DN1). Data are presented as average ± SEM (n>8). L denotes left brain hemisphere, R denotes right brain hemisphere. Navy (8d)/red (80d) bars denote ventral lateral neurons, blue (8d)/orange (80d) bars denote dorsal lateral neurons, and green (8d)/magenta (80d) bars denote dorsal neuron group 1.


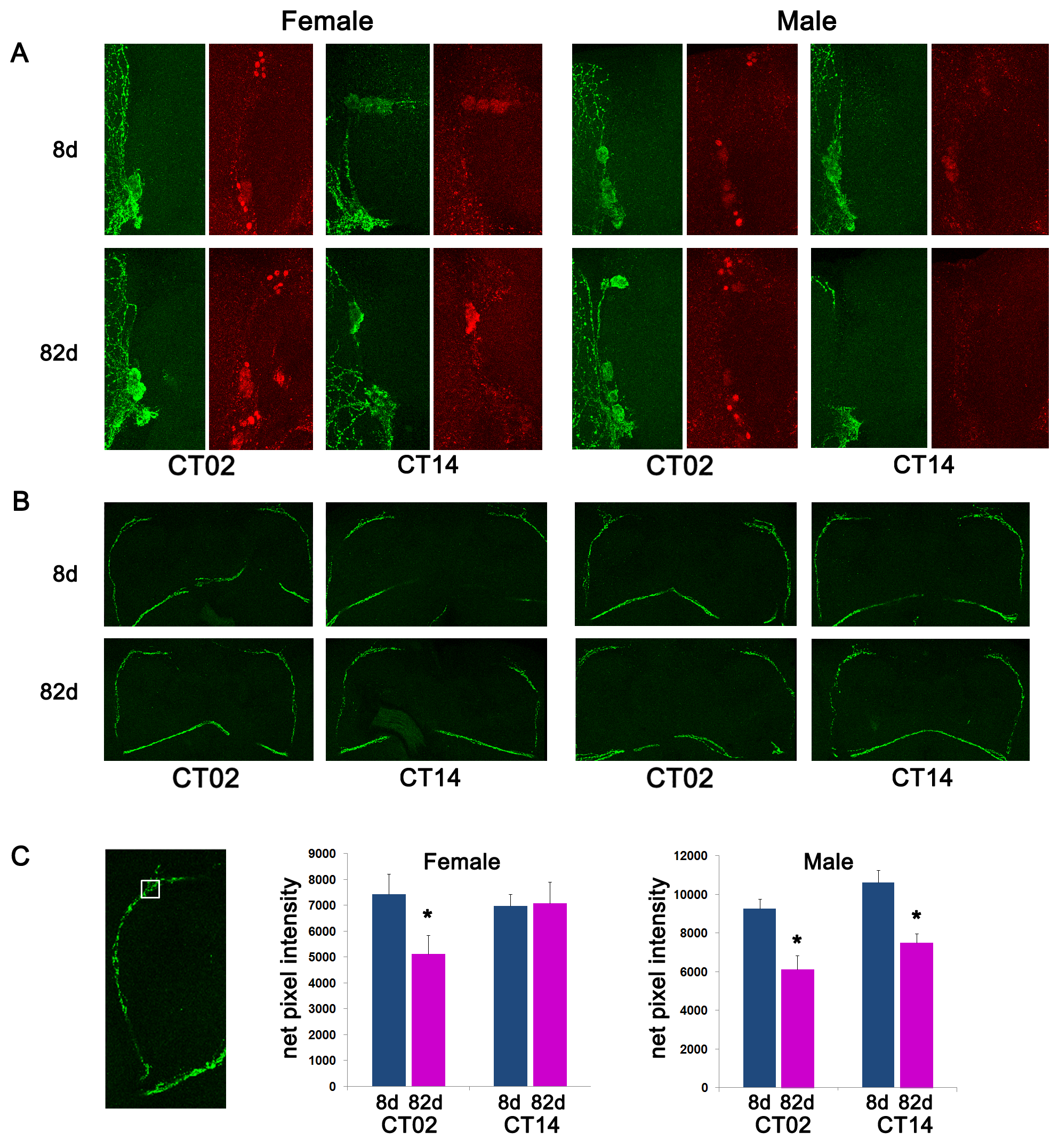


Figure S8. PDF expression in aged flies. (A and B) Young (8d) and arrhythmic terminal age (82d) flies show similar overall levels of PDF (in green) expression in the ventral lateral neurons (panel A, also indicated by PER in red) and dorsal projections (panel B). Old flies were monitored for circadian behavior and arrhythmic flies were examined for PDF expression on days 3 in constant darkness, along with young control flies. The posterior optic tract that connects lateral neurons in the two brain hemispheres and the dorsal projection from lateral neurons are clearly visible in both young and aged flies (panel B). In general, PDF expression is intact in the aged flies at both CT02 and CT14. (C) Quantification of PDF immune-staining intensity at the terminus of the dorsal projection from lateral neurons. Net pixel intensity is quantified for the white square area near the first ramification fork of the dorsal terminal using Kodak Molecular Imaging program. Data are presented as mean ± standard error of mean. *P<0.05, by Student’s T-test assuming unequal variance.


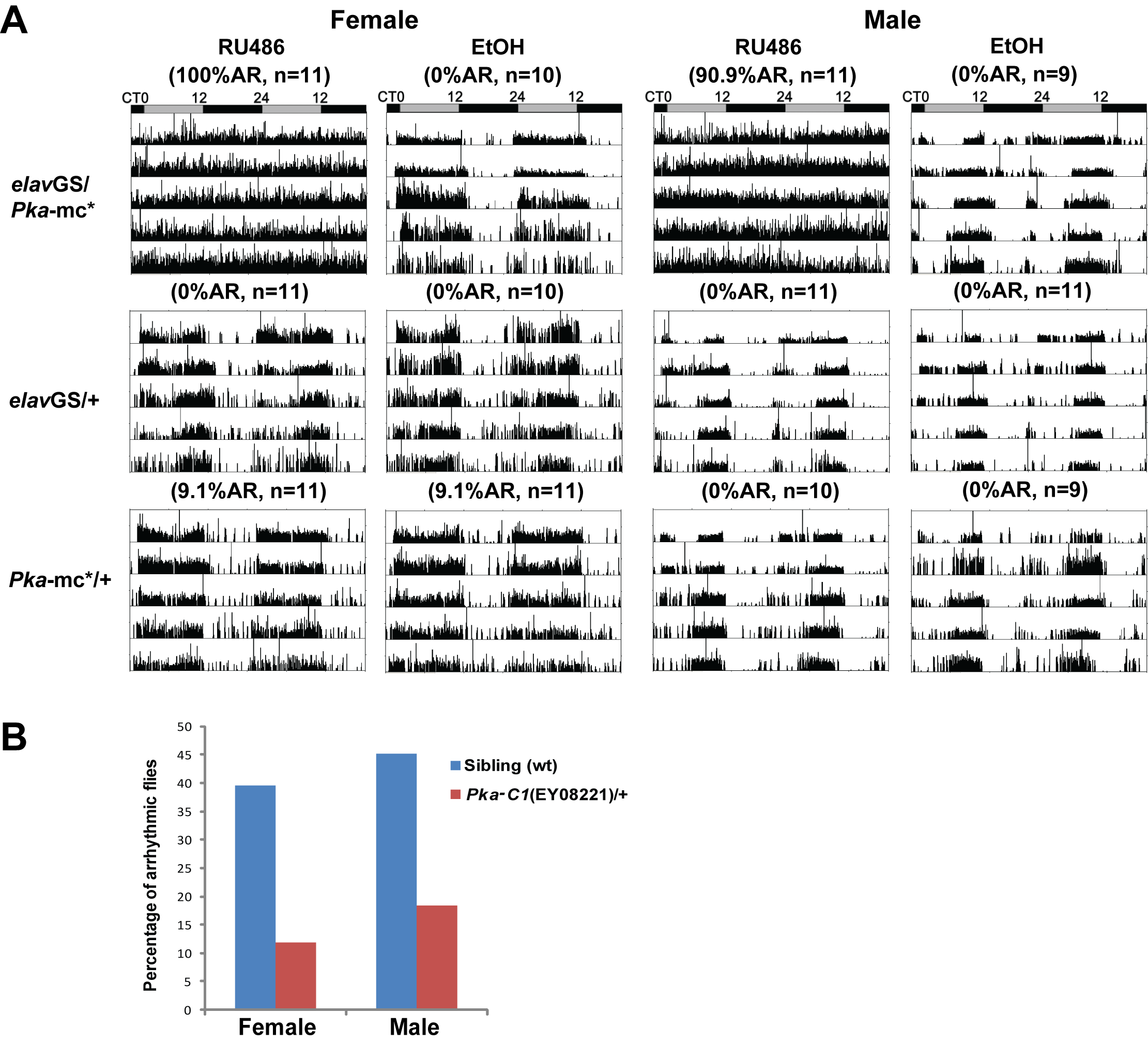


Figure S9. Increasing PKA signaling in young flies causes arrhythmia, while a *Pka-C1* mutant allele partially rescues the arrhythmic phenotype of old flies. (A) Elevation of PKA signaling through over-expression of the PKA catalytic subunit (*Pka-mc**) in the adult nervous system with a drug-inducible *elav*GeneSwitch (*elav*GS) driver, led to arrhythmia in most flies in DD. For drug treatment, flies were reared on regular food and then young adults were maintained on 500μM progesterone (RU486) or vehicle control (ethanol) in LD for 3 days and then transferred to DD. *Gal4* or UAS transgene alone serves as wild-type controls. (B) A *Pka-C1*^EY08221^/+ mutant partially rescues the arrhythmic phenotype of 60 days old flies. Compared to sibling controls, the percentages of flies showing arrhythmic behavior in DD are reduced in *Pka-C1*^EY08221^/+ mutants. This mutant allele was outcrossed five times into an iso31 background and progenies were aged for 60 days in LD. Data were collected for more than 5 days. Sample numbers are: female sib (n=48) and *Pka-C1*^EY08221^/+ (n=67), male sib (n=51) and *Pka-C1*^EY08221^/+ (n=82).


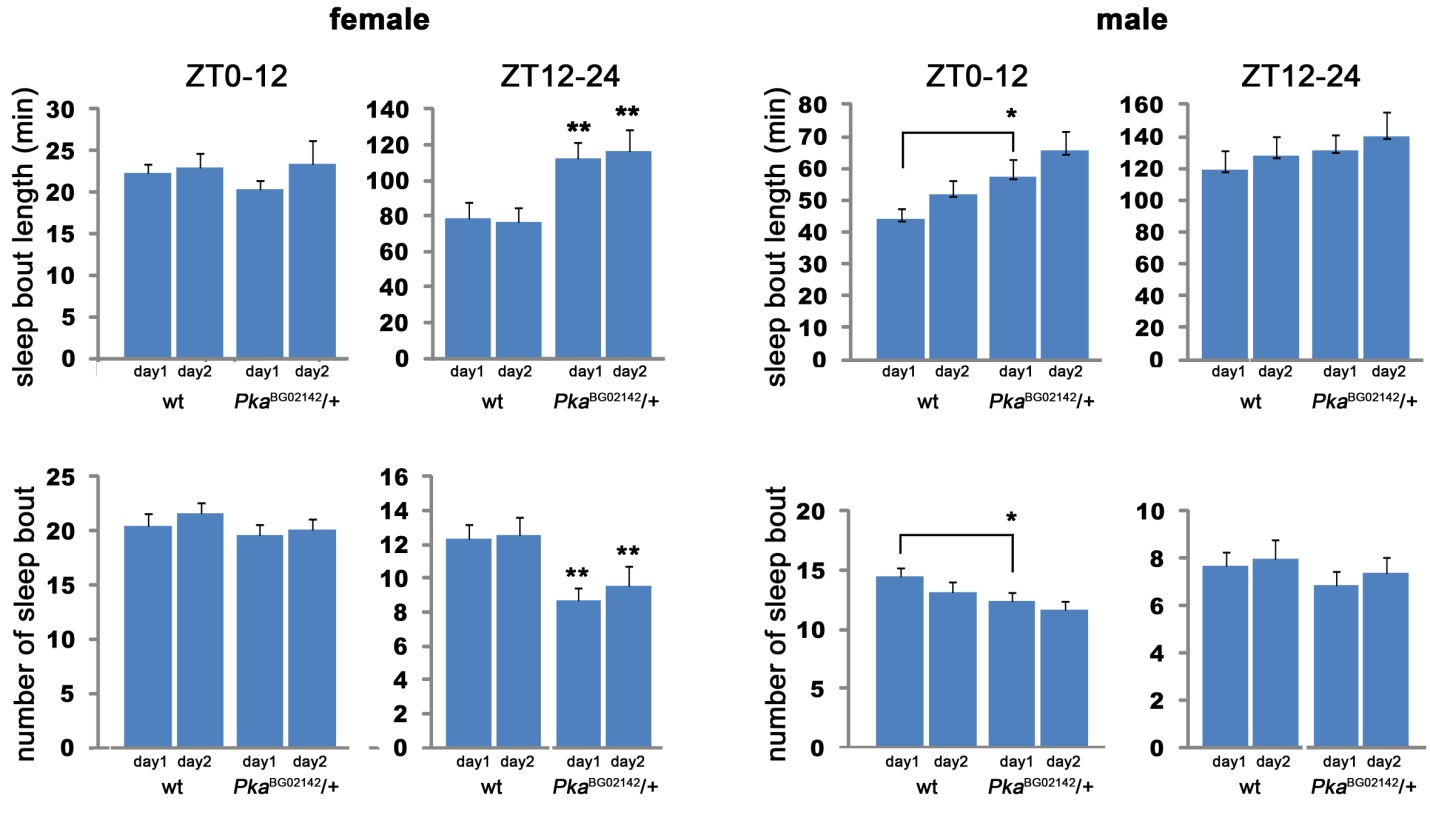


Figure S10. Reducing PKA expression improves sleep maintenance in aged flies. Compared to sibling controls (wt), female *Pka-C1*^BG02142^/+ mutant flies have increased sleep bout length during night which is accompanied by reduced sleep bout numbers. Male flies display similar trend but with smaller effect. 55-day-old flies were monitored under LD condition and locomotor activity data was collected in 1 min bins for sleep analysis. Data are presented as mean ± SEM. Sample numbers are: female wt (n=58) and *Pka-C1*^BG02142^/+ (n=54), male wt (n=49) and *Pka-C1*^BG02142^/+ (n=56). *P<0.05, **P<0.01, by Student’s T-test.
